# Specific Relapse Predictors: Could Cognitive-Behavioral Treatment for Smoking Cessation Be Improved?

**DOI:** 10.3390/ijerph17124317

**Published:** 2020-06-17

**Authors:** Carmela Martínez-Vispo, Ana López-Durán, Carmen Senra, Elisardo Becoña

**Affiliations:** 1 Smoking Cessation and Addictive Disorders Unit, Department of Clinical Psychology and Psychobiology, Faculty of Psychology, University of Santiago de Compostela, 15782 Santiago de Compostela, Spain; 2 Department of Clinical Psychology and Psychobiology, Faculty of Psychology, University of Santiago de Compostela, 15782 Santiago de Compostela, Spain; ana.lopez@usc.es (A.L.-D.); carmen.senra@usc.es (C.S.); elisardo.becona@usc.es (E.B.)

**Keywords:** relapse, smoking cessation, cognitive-behavioral treatment, post-treatment variables

## Abstract

Relapse remains a frequent and complex phenomenon that is not yet well understood. An under-researched area of study that may provide relevant information concerns the assessment of specific post-treatment variables, rather than the composite measures commonly used to predict smoking relapse. The current study sought to examine the effects of post-treatment smoking-related variables, including withdrawal symptomatology, abstinence self-efficacy, and smoking urgency in negative-affect situations and smoking relapse at the 3 month follow-up. The sample comprised 130 participants who achieved abstinence for at least 24 h through a cognitive-behavioral smoking cessation treatment. Regression analysis was conducted for both composite measures and specific subscales and items. Data showed that composite measures of tobacco withdrawal, self-efficacy, and smoking urgency in negative-affect situations were not significant predictors of smoking relapse. However, the analysis including subscales, and specific items showed that lower self-efficacy in negative-affect-related situations (OR = 1.36) and three withdrawal symptoms—irritability/frustration/anger (OR = 2.99), restlessness/impatience (OR = 1.87), and craving (OR = 2.31)—were significant predictors of relapse. These findings offer new insights into the role of different smoking-related post-treatment variables in short-term relapse. Considering and specifically targeting these variables after achieving abstinence may potentially contribute to reducing smoking relapse.

## 1. Introduction

Tobacco use constitutes the major cause of preventable death and disability worldwide and is considered a significant public health concern [1]. Although smoking cessation interventions are shown to be efficacious [2,3], smoking relapse is a frequent and challenging phenomenon. Studies report that a high percentage of smokers receiving smoking cessation treatment and achieving abstinence relapse in the following weeks and months [4,5], with relapse rates ranging approximately from 30% to 80% [6,7].

Research has tried to identify variables predicting smoking relapse [8], including, among others, cigarette consumption per day, years smoking, living with a smoker, tobacco dependence [9], tobacco withdrawal [10], craving [11], stage of change [12], smoking self-efficacy [13], negative affect [14], and even sociodemographic variables such as age, sex, or education [15]. Among the most robust factors related to a higher risk of relapse are withdrawal symptoms [10,16]. Such symptoms are aversive and especially relevant during the first weeks of abstinence [17]. Among them are included depressed mood/sadness, irritability, frustration, anxiety, anger, difficulty concentrating, restlessness/impatience, sleep-related problems, increased appetite, or weight gain [17,18], which are usually assessed together as a composite score. However, there is evidence that withdrawal symptomatology is highly variable between subjects [19] and, therefore, the use of a composite measurement may overshadow the impact of individual withdrawal symptoms on smoking relapse. Negative affect has also been identified as a motivational factor of smoking behavior [20], as individuals tend to experience increased smoking urgency in this type of situation. In fact, some studies have reported that negative affect increases during quit attempts [21] and is associated with a higher likelihood of relapse [14,22].

Another significant variable involved in smoking relapse is self-efficacy, defined as confidence in one’s ability to quit or avoid smoking [23]. This variable has been found to be associated with smoking lapse and relapse during unaided quit attempts [24] and after receiving a smoking cessation intervention [13]. Besides, it has been suggested that abstinence self-efficacy should be examined across different situations, as confidence in maintaining abstinence may differ in specific situations [23]. For instance, in a cross-sectional study examining smoking cessation predictors in a sample of Chinese participants [25], the authors found that self-efficacy related to negative-affect situations was significantly associated with successful smoking cessation, whereas positive affect and habit-related self-efficacy did not reach statistical significance. These results highlight the relevance of considering specific contextual aspects of the smoking self-efficacy construct.

In a smoking cessation treatment context, most studies have evaluated the aforementioned variables at pre-treatment. However, most smoking-related variables could be considered as dynamic [26], which implies that they may change due to the fact of quitting smoking itself (i.e., withdrawal symptoms) or because of the cognitive-behavioral strategies used during the intervention (i.e., self-efficacy). Therefore, examining the effect of the variables that are modified by abstinence achievement and treatment components could contribute to a better understanding of which ones could hinder abstinence maintenance.

Although extensive research has been conducted on smoking relapse, to our knowledge, no studies have compared the differential predictive value between composite measures and their corresponding subscales or specific elements in the relapse of smokers who received cognitive-behavioral treatment for smoking cessation. Therefore, the aims of the study were (1) to examine the effect of post-treatment tobacco withdrawal symptomatology, smoking-related self-efficacy, and post-quit smoking urgency in negative-affect situations using a composite measure on smoking relapse; and (2) to determine whether the use of their specific subscales or items provides additional information compared with the composite scores.

## 2. Materials and Methods

### 2.1. Participants

The original sample was composed of 219 smokers enrolled in a randomized clinical trial for smoking cessation (NCT Identifier: 02844595). For the purpose of this study, we selected those participants who achieved abstinence at the end of the intervention (*n* = 130). The present study is a post hoc analysis of this trial, and a complete description was previously published [27,28]. To be included in the study, participants had to be 18 years of age or older, be current daily smokers (at least 8 cigarettes per day), wish to participate in the treatment program, and provide written informed consent. The exclusion criteria were a diagnosis of severe mental disorder or other substance use disorder; having received a cognitive-behavioral or pharmacological smoking cessation treatment over the previous year; having a high-life-risk disease, or using tobacco products other than cigarettes.

### 2.2. Instruments

Assessments were conducted at pre-treatment (before the beginning of treatment sessions) and post-treatment (during the last treatment session after eight weeks). Self-report data included demographics and smoking-related variables. The following questionnaires were used:

At pre-treatment:

Smoking Habit Questionnaire [29]. This instrument consists of 56 items assessing sociodemographics and smoking-related variables (i.e., years smoking, living with a smoker).

Fagerström Test of Cigarette Dependence (FTCD) [30,31]. This six-item instrument assesses cigarette dependence, with higher scores indicating greater cigarette dependence (i.e., How soon after you wake up do you smoke your first cigarette?). Cronbach alpha was 0.65 in this sample.

At post-treatment:

The Minnesota Nicotine Withdrawal Scale (MNWS) [32]. This instrument assesses eight specific tobacco withdrawal symptoms on a scale ranging from 0 (*absent*) to 4 (*severe*): irritability/frustration/anger, anxiety, difficulty concentrating, restlessness/impatience, increased appetite/hungry/weight gain, depressed mood/sadness, insomnia/sleep problems/awakening at night, and desire/craving to smoke. Cronbach alpha was 0.82 in the present sample.

The Negative Affect Scale of the Tobacco Motivation Questionnaire (NAS-TMQ) [33]. This instrument specifically assesses smoking urges in negative-affect situations (i.e., urgency to smoke when feeling irritated; urgency to smoke when I’m worried about possible misfortunes). This instrument has two subscales: Smoking Urgency in Anxiety-related situations and Smoking Urgency in Anger-related situations. In the present sample, Cronbach alpha was 0.95 for the composite measure, 0.91 for the Anxiety subscale, and 0.92 for the Anger subscale.

The Smoking Self-Efficacy Scale short form (SSE) [34]. This nine-item instrument is used to measure quitting-related self-efficacy. Concretely, this instrument assesses the participant’s confidence in resisting temptations to smoke-related habit/craving (i.e., when I feel I need a lift), negative affect (i.e., when things are not going my way, and I am frustrated), and positive/social situations (i.e., over coffee while talking and relaxing). Higher scores reflect lower cessation-related self-efficacy. Cronbach alpha was 0.88 in the present sample for the composite score, and 0.65 for the positive-affect situations, 0.89 for the negative-affect situations, and 0.78 for craving/habit situations.

### 2.3. Procedure

After the baseline assessment session, participants were randomized to one of the three trial conditions: (1) a standard smoking cessation cognitive-behavioral treatment (SCBSCT); (2) a cognitive-behavioral smoking cessation treatment with components of behavioral activation (SCBSCT-BA); and (3) a wait-list control group. Both active conditions (1 and 2) were cognitive-behavioral interventions consisting of eight weekly sessions administered in groups of 6–8 participants.

For this study, we have included the participants of both active treatment conditions (1 and 2) who reported being abstinent for at least 24 h during the last intervention session (Week 8) and having a carbon monoxide (CO) level ≤5 ppm [35]. We used the Micro + Smokerlyzer^®^ (Bedfont Scientific Ltd., Sittingbourne, UK) to corroborate self-reported abstinence. Relapse was defined as self-report of smoking seven days before the 3 month follow-up session. Those participants who did not attend the follow-up or reported abstinence but had a CO level of >5 ppm were classified as relapsers [36]. We chose this timeframe because research indicates that the risk of relapse is highest within the first 3 months of the abstinence period [37].

The Bioethics Committee of the University of Santiago de Compostela approved the study, which was conducted in compliance with the Declaration of Helsinki’s ethical principles.

### 2.4. Data Analyses

All statistical analyses were conducted using the SPSS software version 24 (IBM Corp., Armonk, NY, USA). A *p*-value of ≤0.05 was used as a test of statistical significance. Firstly, a descriptive analysis of the overall demographic characteristics and smoking-related variables was performed. Then, comparisons of sociodemographic and pre-treatment smoking-related variables between the abstinent participants and the relapsers were conducted using Student’s *t*-test for continuous variables and the chi-square test for categorical variables.

The predictive value of the study variables on relapse at the 3 month follow-up was assessed using binary logistic regression models. Sociodemographic and pre-treatment smoking-related variables reaching statistical significance at the 0.1 level in the bivariate analyses would be entered as covariates in the multivariable model. Two logistic regression models were conducted. In the first regression model, the composite scores of withdrawal symptoms, self-efficacy, and negative-affect-related smoking urgency measured at post-treatment were included as the predictor variables. In the second regression model, scores of each withdrawal symptom (irritability/frustration/anger, anxiety, difficulty concentrating, restlessness/impatience, increased appetite/hungry/weight gain, depressed mood/sadness, insomnia/sleep problems/awakening at night, and desire/craving to smoke), the three self-efficacy subscales (in positive-affect situations, negative-affect situations, and habit/craving situations), and the two subscales of negative-affect-related smoking urgency (anxiety and anger) were included as the predictor variables. Nagelkerke’s *R*^2^ statistic was used to assess the proportion of total variance explained by each set of variables [38]. The goodness of fit of the regression models was checked via chi-square, using the Hosmer–Lemeshow test [39].

## 3. Results

### 3.1. Descriptive Analysis

Of the total sample, 66 (50.8%) maintained abstinence at the 3 month follow-up, 49 (37.7%) reported having relapsed, and 15 (11.5%) did not provide data. No significant differences were found in demographics and pre-treatment smoking-related variables according to smoking status at the 3 month follow-up (Table 1).

### 3.2. Regression Analyses

Since the bivariate analysis did not yield significant differences between participants who remained abstainers, and those who relapsed (Table 1), the multivariable logistic regression analyses were conducted without including covariates. Results of the first regression model indicated that none of the three post-treatment composite scores of tobacco withdrawal, self-efficacy, and smoking urgency in negative-affect situations were significantly related to relapse at the 3 month follow-up (Table 2).

The second multivariable logistic regression model (Table 3), including each specific subscale and each symptom of tobacco withdrawal, showed adequate goodness of fit (Hosmer–Lemeshow χ^2^ = 8.14, *p* = 0.420). The Nagelkerke pseudo-*R*^2^ indicated that 31.6% of the variance in smoking relapse was accounted for by the overall predictors. In this model, the variables that were significant predictors of smoking relapse at the 3 month follow-up were lower self-efficacy in negative-affect situations (OR = 1.36), and three specific withdrawal symptoms: irritability/frustration/anger (OR = 2.99), restlessness/impatience (OR = 1.87), and craving (OR = 2.31).

## 4. Discussion

The present study compared the predictive value of the composite measures of post-treatment self-efficacy, smoking urgency in negative-affect situations, and tobacco withdrawal, as well as the specific predictive value of such variables, but using their subscales or their items, on smoking relapse at the 3 month follow-up in a sample of smokers who quit smoking after attending a cognitive-behavioral smoking cessation treatment.

Results showed that there were no significant differences between relapsers and abstinent participants in sociodemographic variables, pre-treatment smoking-related variables, or treatment condition received. When considering post-treatment smoking-related variables’ composite measures, none of the study variables were significantly related to smoking relapse. However, when examining the regression model including each specific subscale and item, our data showed that lower post-treatment smoking-related self-efficacy in negative-affect situations was a significant predictor of smoking relapse. Concretely, these findings are in line with previous research [25], highlighting the importance of considering specific self-efficacy-related contextual aspects, as a global measure of confidence in one’s ability to abstain may not capture the relevance of situational factors that impact on smoking cessation outcomes [23].

Concerning the withdrawal syndrome, only three specific symptoms (irritability/frustration/anger, restlessness, and craving) showed a significant relation with smoking relapse. Following the previous literature, our results are consistent with the idea that individual symptoms are meaningful in their own right [40] and with previous research finding that such symptoms are strongly related to smoking relapse during the first months of abstinence [41].

Contrary to our expectations, this study showed that post-treatment smoking urgency in negative-affect situations (anger and anxiety) did not predict smoking relapse at the 3 month follow-up. Some of the strategies trained during the smoking cessation treatment may be influencing this result, as participants could have learned to cope with smoking urgency in such situations. It is also plausible that smoking urgency itself is not enough to predict relapse, and that other variables may interact with it, such as, for example, individual impulsivity [42] or cessation fatigue [43]. Further research is needed to examine the impact of this variable on smoking relapse.

The findings of this study may have significant implications for smoking cessation treatments. Our data showed that the relapse predictors in this study are potentially modifiable, and therefore, could be considered intervention targets. For instance, lower self-efficacy in negative-affect situations at the end of treatment, as well as greater negative-affect withdrawal symptoms and craving, could be addressed by incorporating intermediate sessions between the final treatment session and the 3 month follow-up, in which participants could learn strategies to cope with these specific difficulties. Another possibility could be to incorporate technology-based strategies to support and maintain contact with participants after treatment completion during the follow-up period. Such technology-based strategies (i.e., tailored mobile phone messaging, social media support groups) could be used to aid participants to cope with the distress associated with quitting as well as with their confidence to be able to manage smoking urges in specific high-risk contexts or situations.

This study has several limitations that should be considered in the interpretation of results. First, the current sample comprised treatment-seeking smokers who attended a cognitive-behavioral treatment to quit smoking, and therefore, findings are not generalizable to smokers achieving unaided abstinence. Second, the current study focused exclusively on demographics and smoking-related variables. Because other psychological factors could be related to smoking relapse, such as individual cue reactivity or coping skills [44,45], future research should analyze the contribution of such variables in the complex process of smoking relapse. Third, study variables were measured through self-report instruments, and consequently, could be influenced by response bias. Lastly, we only considered withdrawal symptoms measured through the MNWS. Recent research has suggested that symptoms such as anhedonia [46] or mood swings [47] should be considered among withdrawal symptoms as well, and therefore, future studies should include such variables when examining the impact of withdrawal symptoms on relapse.

## 5. Conclusions

In conclusion, findings of the present study highlight the complexity of factors impacting on smoking relapse and contribute to the existing literature by identifying relevant variables that should be targeted during smoking cessation treatments, particularly, during the final phase of smoking cessation interventions or after treatment completion. The identification of participants with higher scores of such variables at post-treatment could imply the possibility of giving them extra aid to minimize and prevent smoking relapse. Future research is warranted to examine whether including therapeutic strategies that address these variables during the first weeks and months of abstinence could reduce smoking relapse rates.

## Figures and Tables

**Table 1:** Sociodemographic and smoking-related variables according to smoking status at the 3 month follow-up.

Variables	Relapse (*n* = 64)	Abstinence (*n* = 66)	χ^2^/t	*p*
	M (SD)/% (*n*)	M (SD)/% (*n*)		
Age	45.03 (10.98)	44.68 (11.09)	−0.180	0.857
Sex (female)	68.8 (44)	63.6 (42)	0.379	0.538
Marital status (married/living with a partner)	45.6 (31)	54.4 (37)	0.757	0.384
Education (University)	39.1 (25)	43.9 (29)	0.318	0.573
Pre-treatment CO	19.31 (10.52)	17.23 (6.70)	−1.343	0.182
Cigarettes per day	18.64 (5.71)	17.12 (6.12)	−1.464	0.146
Years smoking	26.22 (11.38)	26.15 (11.24)	−0.034	0.973
Living with a smoker (yes)	34.4 (22)	36.2 (24)	0.056	0.813
Treatment condition (SCBSCT-BA)	53.1 (34)	62.1 (41)	1.077	0.299
FTCD	4.51 (1.97)	4.23 (1.99)	−0.827	0.410

CO = carbon monoxide; SCBSCT-BA = standard cognitive-behavioral smoking cessation treatment plus behavioral activation; FTCD = Fagerström Test of Cigarette Dependence.

**Table 2:** Binary logistic regression analysis of composite measures predicting smoking relapse at the 3 month follow-up.

Predictors	Exp (*B*)	95% CI	*p*
NAS-TMQ	0.95	0.88–1.02	0.181
SSE	1.08	0.89–1.39	0.058
MNWS	1.05	0.97–1.12	0.187

NAS-TMQ = Negative Affect Scale of the Tobacco Motivation Questionnaire; SSE = Smoking Self-Efficacy Scale; MNWS = Minnesota Nicotine Withdrawal Scale, CI = confidence interval.

**Table 3:** Binary logistic regression analysis of subscales and specific symptoms predicting smoking relapse at the 3 month follow-up.

Predictors	Exp (*B*)	95% CI	*p*
NAS-TMQ Anxiety	0.91	0.73–1.12	0.374
NAS-TMQ Anger	0.88	0.74–1.04	0.149
SSE positive affect	1.07	0.89–1.28	0.497
SSE negative affect	1.36	1.06–1.74	0.015
SSE craving/habit	0.86	0.69–1.06	0.161
MNWS 1 Irritability/frustration/anger	2.99	1.48–6.05	0.003
MNWS 2 Anxiety	1.33	0.73–2.44	0.351
MNWS 3 Difficulty concentrating	1.09	0.63–1.91	0.751
MNWS 4 Restlessness	1.87	1.01–3.48	0.049
MNWS 5 Increased appetite/weight gain	0.82	0.57–1.19	0.299
MNWS 6 Insomnia/sleep problems	1.09	0.76–1.55	0.634
MNWS 7 Depressed/sad mood	0.77	0.42–1.30	0.303
MNWS 8 Craving for cigarettes	2.31	1.39–3.83	0.001

NAS-TMQ = Negative Affect Scale of the Tobacco Motivation Questionnaire; SSE = Smoking Self-Efficacy Scale; MNWS = Minnesota Nicotine Withdrawal Scale, CI = confidence interval.
